# Ethno-ethics Lens for Palliative Care Decision-making in COVID-19

**DOI:** 10.30476/ijcbnm.2021.89239.1713

**Published:** 2021-07

**Authors:** Karolus Yosef Woitila Wangi

**Affiliations:** Department of Nursing, Tarumanagara School of Health Sciences, Jakarta, Indonesia

## DEAR EDITOR

The Coronavirus Disease (COVID-19), which emerged in late 2019, has spread throughout the world as a widely known pandemic, claiming over 3 million lives as of April 2021. ^1^ The chaos resulting from the spread of this infectious disease caused great uncertainty and an urgent need to find an effective treatment. However, the issue in handling COVID-19 patients is limiting the number of patient care services available. During the COVID-19 pandemic, palliative care has received significant public attention. ^2^ The COVID-19 patients with certain conditions who do not meet Intensive Care Unit criteria can alternatively expect palliative care. Palliative care is handled by the Hospital Palliative Care Team, which offers decision-making support and psychosocial care for patients and their families. ^3^

Decision-making in palliative care has proven to be complicated because of the uncertain prognosis and general fears surrounding the decision. This indirectly creates ethical dilemmas for patients, families, and health care workers. Spiritual and cultural aspects can influence this decision-making. By providing spiritual support and special care with consideration of the patient’s values and beliefs during the illness, patients can make earlier decisions and have shown lower conflict decision-making and greater satisfaction with one’s decisions. ^4^ Spiritual support would be beneficial and have a positive impact on ethical decision-making in inpatient care.

According to the research of the history of global and multicultural societies in the 1980s, anthropological researchers have brought light to universalistic-nature ethical principles. ^5^ In other words, these principles must be analyzed and acculturated with the local culture so that they can accommodate potential local issues to produce derivative principles. This concept also takes into account the four broad moral principles that existed to be the basis of modern biomedical ethics, entitled “Principlism”, which consists of respect for person (autonomy), non-maleficence, beneficence, and justice. ^6^

Palliative care, as a global issue, has been accommodated by the World Health Organization (WHO). In 2018, WHO released guidance on integrating palliative care and symptom relief into responses to humanitarian emergencies and crises. The elaboration shows that several ethical principles are relevant to be applied in the COVID-19 pandemic within the framework of “Principlism” to accommodate the issue of diversity in ethno-culture including:

1. Respect for person (autonomy): All patients’ dignity and human rights must be respected, and health professionals should provide them with all health-related information, respect their decision-making, and provide appropriate recommendations;

2. Non-maleficence: All patients should have access to palliative care to minimize suffering and eradicate discrimination based on ethnicity, religion, gender, age, or political affiliation;

3. Beneficence: The patients or family may have conflict with the public good such as infectious disease, so that the great judiciousness must be shown;

4. Justice: Similar patients should be treated similarly regardless of ethnicity, religion, gender, age, or political affiliation. ^2^

In conclusion, in response to the COVID-19 pandemic, health workers are faced with the decision to comply in accordance with the latest patient handling procedures. If health care workers have made the best efforts, but the patient has experienced a poor prognosis, palliative care needs to be the most potential alternative. The patient must be accommodated with an informed choice so that patients with different cultural backgrounds can consider and make the best decisions for themselves and consciously accept the palliative care decisions that have been taken. Ethical principles in the framework of “Principlism” in a humanitarian crisis use an ethno-ethics lens to oversee potential ethical issues surrounding decision-making in palliative care. Health care workers must sensitively use these ethical guidelines to accommodate the ethno-culture backgrounds of various patients. This global policy needs to be translated into each country’s national policies by elaborating the framework of “Principlism” to produce derivative rules that accommodate local ethical issues in various dilemma situations with COVID-19 patients.
